# Laparoscopic restorative proctocolectomy with ileal-J-pouch anal canal anastomosis without diverting ileostomy for total colonic and extensive aganglionosis is safe and feasible with combined Lugol's iodine staining technique and indocyanine green fluorescence angiography

**DOI:** 10.3389/fped.2022.1090336

**Published:** 2023-01-06

**Authors:** Yoichi Nakagawa, Kazuki Yokota, Hiroo Uchida, Akinari Hinoki, Chiyoe Shirota, Takahisa Tainaka, Wataru Sumida, Satoshi Makita, Hizuru Amano, Aitaro Takimoto, Seiya Ogata, Shunya Takada, Takuya Maeda, Yousuke Gohda

**Affiliations:** ^1^ Department of Pediatric Surgery, Nagoya University Graduate School of Medicine, Nagoya, Japan; ^2^ Department of Gastrointestinal and Pediatric Surgery, Mie University Graduate School of Medicine and Faculty of Medicine, Mie, Japan; ^3^ Department of Rare/Intractable Cancer Analysis Research, Nagoya University Graduate School of Medicine, Nagoya, Japan

**Keywords:** total colonic aganglionosis, extensive aganglionosis, laparoscopic restorative proctocolectomy, j pouch, diverting ileostomy, minimally invasive surgery

## Abstract

**Background:**

We present the surgical technique and outcomes of reduced-port laparoscopic restorative proctocolectomy with ileal-J-pouch anal canal anastomosis (IPACA) without diverting ileostomy for total colonic and extensive aganglionosis (TCA+).

**Methods:**

We retrospectively reviewed TCA+ cases between 2014 and 2022. Preoperative ileostomy was performed when transanal bowel irrigation was ineffective. Radical surgery for TCA+ was performed at approximately 6 kg. The surgery was performed using laparoscopy through a multi-channel trocar with or without an additional 3-mm trocar and IPACA reconstruction with indocyanine green fluorescence angiography (ICG) to assess anastomotic perfusion and Lugol's iodine staining to visualize the surgical anal canal.

**Results:**

Ten patients with TCA+ were included. Ileostomy was performed in seven cases. The median operation time and blood loss were 274.5 min and 20 ml, respectively. No significant postoperative complications were found. All patients experienced frequent liquid stools and perianal excoriation in the early postoperative period, requiring anti-flatulence or codeine. The median follow-up period was 3.5 years. Three patients required irrigation management 1 year postoperatively, and the others defecated a median of 3.5 times per day. The median Kelly's clinical score was 5 in 5 patients aged >4 years.

**Conclusion:**

Reduced-port surgery, combined with Lugol's iodine staining and ICG, was safe, feasible, and had cosmetically and clinically acceptable mid-term outcomes.

## 1. Introduction

Total colonic and extensive aganglionosis (TCA+) is a severe form of Hirschsprung disease (HD) that accounts for 9% of all HD cases (1). It is characterized by the absence of ganglion cells throughout the colon, extending into the terminal ileum. Various procedures for TCA+ treatment have been developed; however, the optimal surgical procedure remains unknown. We performed laparoscopic restorative proctocolectomy (RPC) with ileal J-pouch anal canal anastomosis (IPACA) in patients with TCA+. Furthermore, minimally invasive surgery has become mainstream in pediatrics. Although long-term postoperative outcomes are essential, cosmetic outcomes are also important. Therefore, we performed laparoscopic RPC-IPACA using reduced ports. We also used Lugol's staining technique to identify the upper margin of the anal canal and indocyanine green (ICG) fluorescence angiography to assess anastomotic perfusion, which contributed to an adequate anastomosis site and prevented leakage. Here, we present our surgical procedure for treating TCA+ and evaluate its outcomes.

## 2. Materials and methods

We retrospectively reviewed TCA+ cases treated at our institution between 2014 and 2022. All patients were diagnosed with HD by pathological evaluation of rectal or intraoperative biopsy in cases that required enterostomy. Preoperative ileostomy was performed when the intestine was perforated or extremely distended and when preoperative management of bowel irrigation using a trans-anal tube was ineffective. Radical surgery for TCA+ was performed at approximately 6 kg. Radical surgery was performed using laparoscopic RPC-IPACA without a diverting ileostomy. Patient background, surgical results, complications, postoperative management, and bowel function were evaluated retrospectively.

### 2.1. Surgical procedure

**Figure 1:**
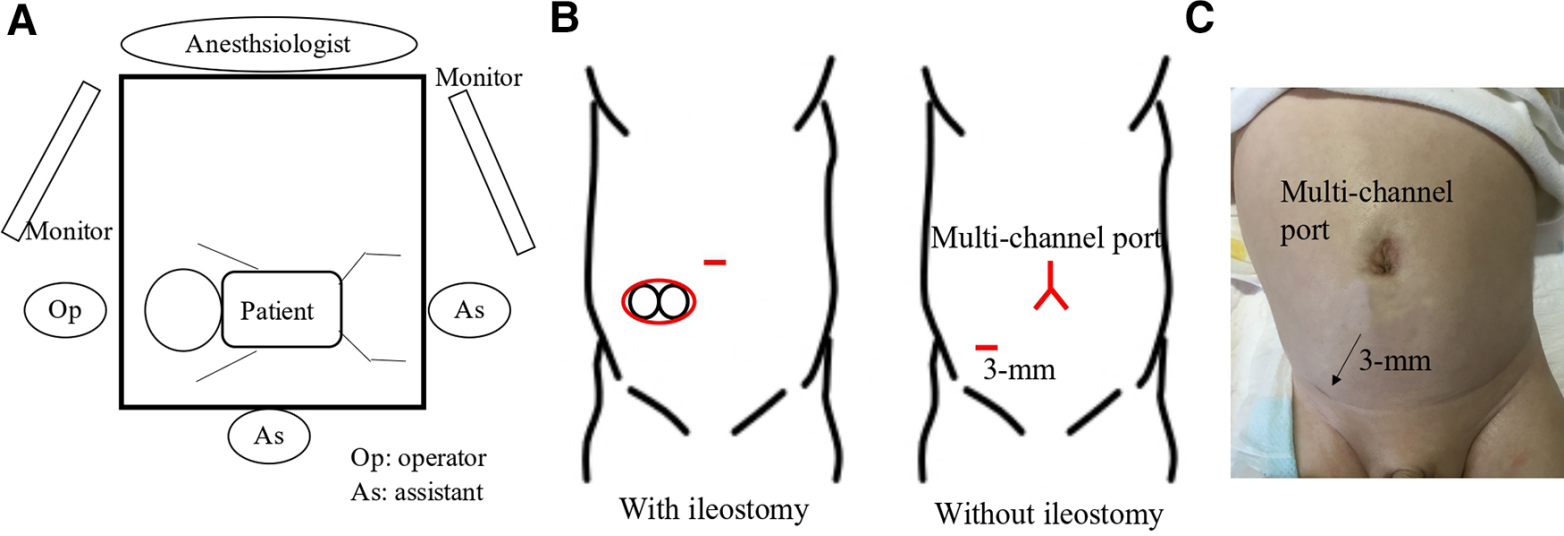
(**A**) the surgeon stood adjacent to the patient's head, and the surgical and camera assistants were positioned on the right and foot side of the patient until the transanal procedure. The surgeon was positioned at the foot side of the patient during the transanal procedure. (**B**) A spindle-shaped incision around the stoma or Benz incision at the umbilicus was made in patients with or without enterostomy, respectively. An additional 3-mm trocar was placed if required. (**C**) The wound 1 month postoperatively is healing correctly and is not so visible.

Under general anesthesia, the patients underwent lithotomy to allow access to both the abdomen and perineum. The anesthesiologist stood on the left side of the patients, while the surgeon stood adjacent to their head, and a monitor was positioned at the patients' feet (Figure 1A).

#### 2.1.1. J-pouch creation

**Figure 2:**
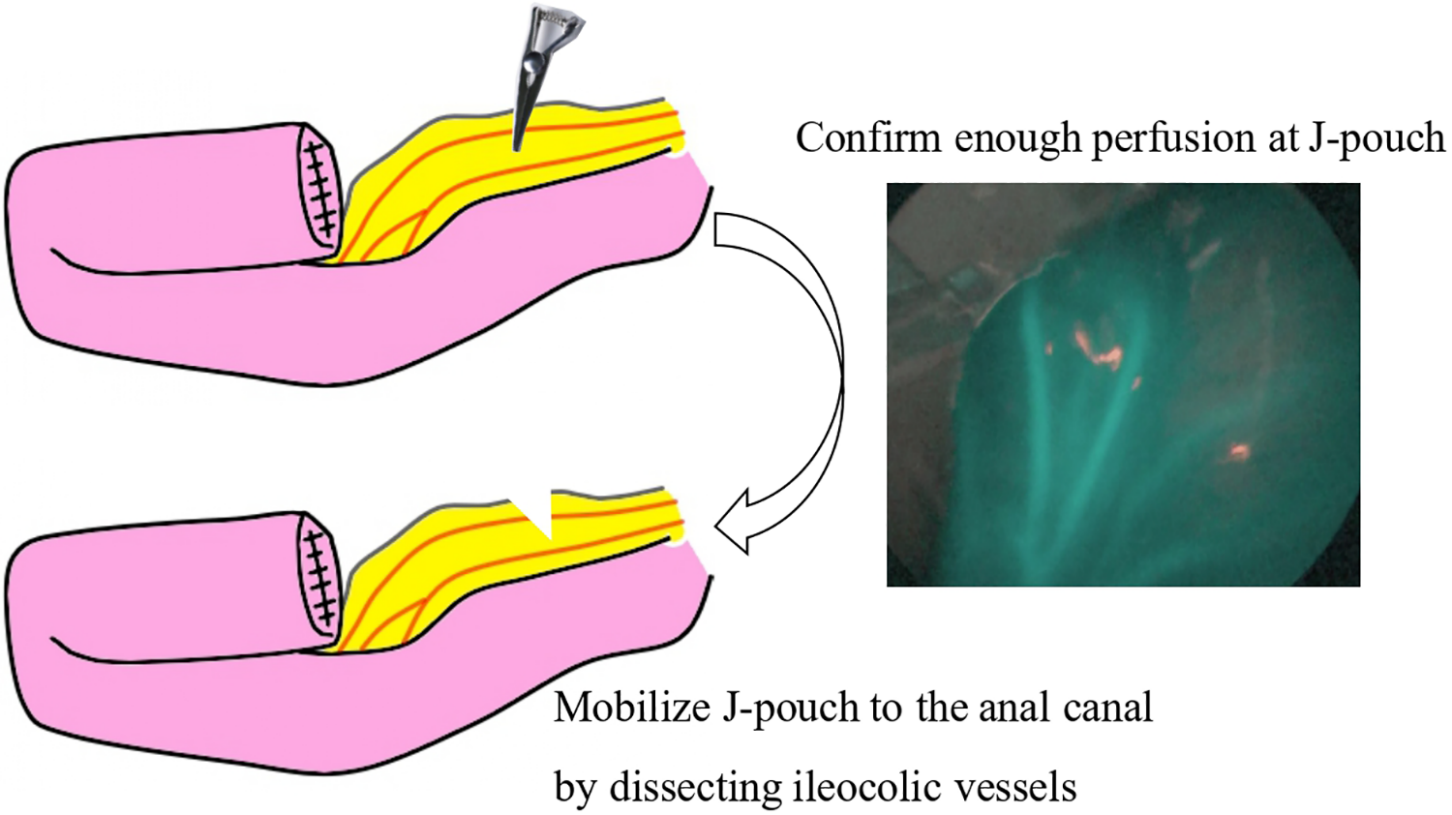
When pulling the J-pouch through the anal canal, it was mobilized by dissecting the mesenchyme after confirming adequate retention for perfusion by indocyanine green fluorescence angiography with a vessel clamp. Indocyanine green was intravenously injected with a dose of 0.01 mg/kg.

A spindle-shaped incision was made around the ileostomy site in patients who underwent ileostomy, and a Benz umbilicus incision (2) was made in patients who did not undergo ileostomy (Figure 1B). A multi-channel access port, such as a free access port (Top, Tokyo, Japan) with an Alexis wound retractor (Applied Medical, Rancho Santa Margarita, CA, United States), was inserted through the incision. Subsequently, three 5 mm trocars were inserted, and a 5 mm 30° scope was inserted through a port. For patients in whom the laparoscopic procedure was difficult, an additional 3 mm trocar was inserted (Figure 1B). The aganglionic bowel from the distal ileum to the sigmoid colon was fully exteriorized through a wound retractor and removed by resecting the mesentery. A J-pouch, which had the ileum folded back for a length of 8 cm–10 cm, was constructed, and the laparoscopic procedure was initiated. The rectum below the peritoneal reflection was dissected circumferentially almost to the level of the internal anal sphincter, which was performed meticulously along the precise lines of the rectal wall until the dissection level reached the internal anal sphincter. When pulling the J-pouch through the anal canal without tension was impossible, it was mobilized by ileocolic vessel ligation at the origin of the superior mesenteric artery, and retention for adequate perfusion was confirmed through ICG fluorescence angiography using a vessel clamp with an intravenous ICG dose of 0.01 mg/kg (Figure 2). We decided whether the perfusion was adequate by observing ICG fluorescence angiography within 1 min after ICG injection.

#### 2.1.2. Trans-anal procedure

**Figure 3:**
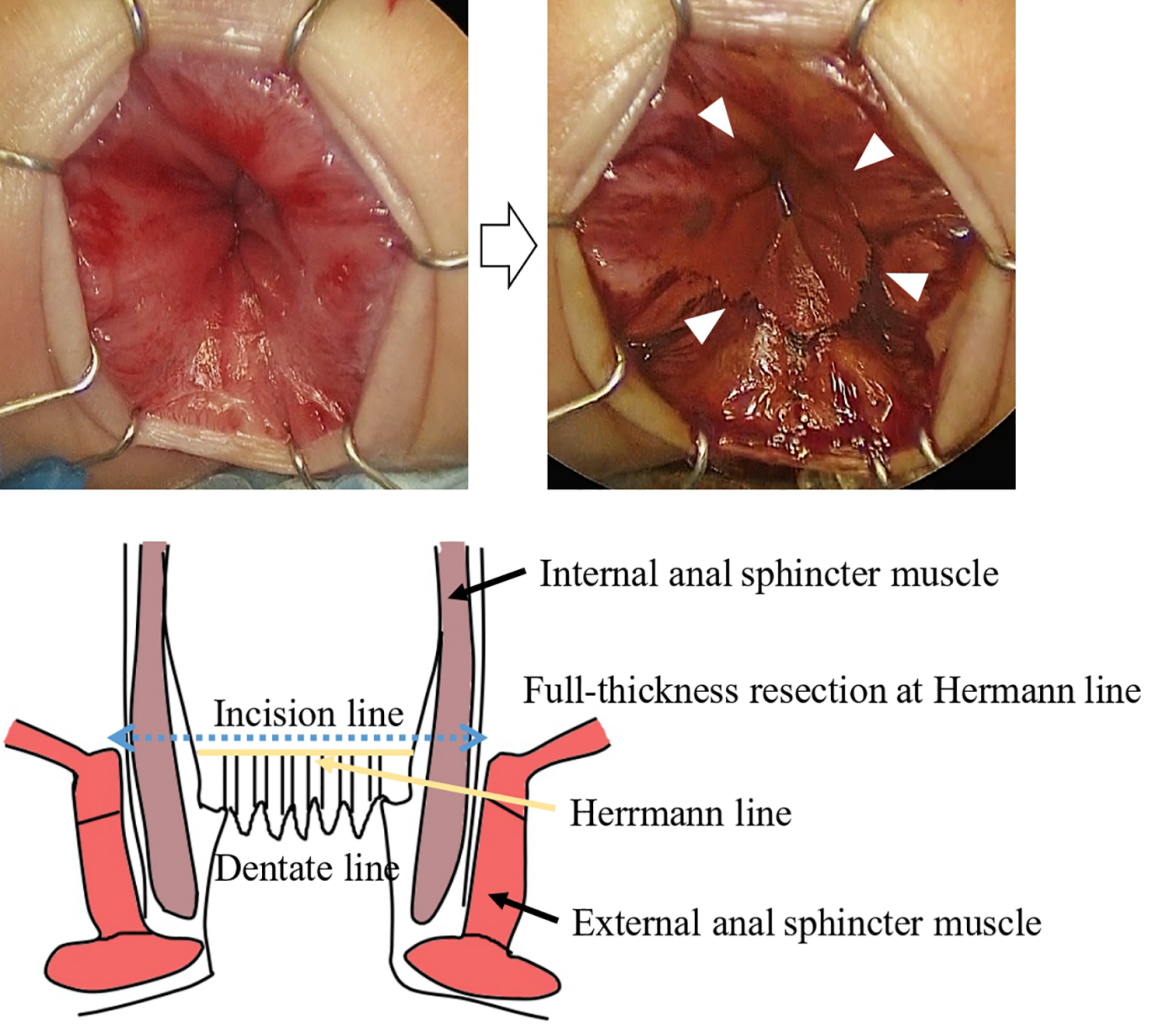
Lugol's iodine staining shows a well-demarcated line indicated by arrowheads (herrmann line). This line allows easy identification of the incision line.

**Figure 4:**
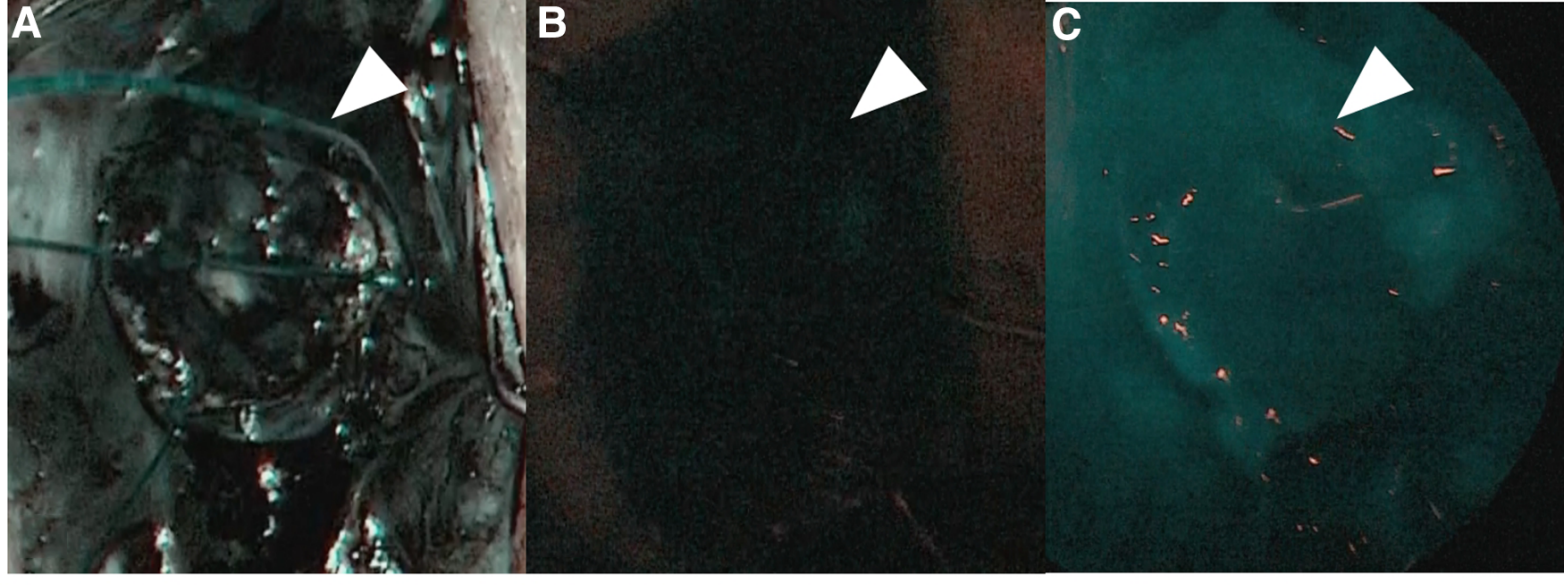
(**A**) Indocyanine green fluorescence angiography was performed to assess the anastomotic perfusion (pre-injection). (**B**) ICG at a dose of 0.01 mg/kg was intravenously injected. (**C**) After confirming the J-pouch with good fluorescence, ileal-J-pouch anal canal anastomosis was constructed.

For this procedure, an anal retractor (Lone Star Retractor System™; Yufu, Tokyo, Japan) was used. Lugol's iodine staining was performed to identify Herrmann's line (3). Briefly, after washing out rectal mucus with normal saline, 5–10 ml of Lugol's solution containing 1% iodine and 2% potassium iodide was sprayed onto the anal canal. Next, full-thickness resection along Herrmann's line, which was shown as a well-demarcated line by Lugol's solution (Figure 3), was performed, and the aganglionic bowel and muscular cuff were removed. Subsequently, the J-pouch was pulled through the anal canal. Finally, ICG was intravenously administered at a dose of 0.01 mg/kg, and ICG fluorescence angiography was performed to assess the anastomotic perfusion (Figure 4). IPACA was constructed, and the abdominal wall was closed.

### 2.2. Ethical approval

This study was performed in accordance with the ethical standards of the 1964 Declaration of Helsinki and its later amendments or comparable ethical standards. Written informed consent was obtained from the legal guardians. This study was approved by the Institutional Review Board of our institution (2022-0207).

## 3. Results

Table 1 shows the patients' backgrounds and outcomes.

**Table 1:** Details of the study population.

**Patient's background**	
Gestational age*	39w1d (36w3d–41w5d)
Body weight at birth*	2819 g (1610–3946 g)
Type of Hirschsprung's disease	
Total colonic aganglionosis, *n* (%)	8 (80%)
Extensive aganglionosis, *n* (%)	2 (20%)
**Preoperative management**	
Stoma, *n* (%)	7 (70%)
Irrigation, *n* (%)	3 (30%)
**Surgery**	
Body weight at radical surgery*	6.67 kg (2.9–11.4 kg)
Age at radical surgery*	5 months old (2–37 months old)
Operation time*	274.5 min (183–404 min)
Blood loss*	20 ml (2–133 ml)
**Postoperative situation**	
Follow up*	3.5 years (0–8 years)
Hirschsprung's disease associated enterocolitis, *n* (%)	3 (30%)
Irrigation management, *n* (%)^b†^	3 (30%)
Bowel movement per day^*†^	3.5 (2–5)
Kelly's clinical score* in five patients aged >4 years	5 (2–6)
Continence*	2 (1–2)
Staining*	1 (0–2)
Sphincter*	2 (1–2)

* median (range).

^†^ in seven patients > one year after surgery.

### 3.1. Patient background

Ten patients were included in this study, and eight were boys. They were mostly full-term births with average newborn weights. Comorbidities included esophageal atresia, congenital central hypoventilation syndrome, and Down syndrome. Ileostomy was performed in 7 of 10 cases, and 3 were preoperatively managed using trans-anal irrigation. Total colonic aganglionosis was observed in 8 of 10 cases, and extensive aganglionosis was observed in 2 cases.

### 3.2. Perioperative complications

All 10 patients underwent laparoscopic RPC-IPACA surgery. The median age and body weight at radical surgery were 5 months and 6.67 kg, respectively. The median operation time and blood loss were 274.5 min and 20 ml, respectively. Additional 3-mm trocars were placed in 7 of 10 cases. No apparent ICG-related complications were observed. All patients had buttock dermatitis due to frequent defecation. The median daily incidences of bowel movements 2 weeks, 1 month, and 3 months after surgery were 8.5 (5–17 times), 10 (5–20 times), and 6 (3–15 times) times, respectively. Most patients were successfully treated with ointment, anti-flatulence, or codeine; however, one patient required irrigation management for buttocks dermatitis. No leaks, dysuria, or other significant complications of Clavien–Dindo classification ≥IIIa were found (4).

### 3.3. Mid- and long-term complications

The median follow-up period was 3.5 years. HD-associated enterocolitis occurred five times in 3 of 10 cases, and 3 cases were <1 year after radical surgery. Among seven cases >1 year after radical surgery, three cases required irrigation at home to control bowel movement; the median incidence of bowel movement was 3.5 times per day. One patient required re-operation, which involved the dissection of the fused septum of the J-pouch; however, no other significant complications were found. The median Kelly's clinical score was 5 (2–6) in 5 patients aged >4 years, and each Kelly's clinical score, including continence, staining, and sphincter, was 2 (1–2), 1 (0–2), and 2 (1–2), respectively.

## 4. Discussion

The mortality rate of TCA+ has dropped from 40.9 to 15.8% (5) in Japan; however, TCA+ treatment remains a challenge for pediatric surgeons. Surgical treatment of HD involves various techniques; however, the optimal procedure has not yet been determined. The Swenson procedure was first reported for HD (6) in 1948, but it had the disadvantage of injuring pelvic structures, particularly when dissecting the anterior rectum from the bladder. Therefore, the Duhamel (7) and Soave (8) procedures were developed and have been widely used since then. Currently, the modified Duhamel procedure is accepted as the best option for patients with TCA+ for long-term function (9); nevertheless, the long-term outcomes of TCA+ treatment are still insufficient, with 47% of patients complaining of continence (10). Residual aganglionic muscular cuffs can lead to recurrent enterocolitis and constipation (11). If the cuff is split during the original surgery, it can cause functional stricture by constriction (12). The Duhamel and Soave procedures retain the aganglionic muscular cuff, which may interfere with bowel movements in the long term. Consequently, a surgical procedure that excludes the aganglionic muscular cuff and avoids injury to the pelvic plexus is desired. RPC-IPACA is a well-established procedure for ulcerative colitis and familial adenomatous polyposis in pediatric patients (13) and is also used for HD (14). We believe that RPC-IPACA is theoretically the best treatment for patients with TCA+ because it can completely resect the aganglionic muscle cuff. Although limited data exist regarding the effect of RPC-IPACA on long-term bowel function in patients with TCA+, laparoscopic RPC-IPACA for 73 patients with ulcerative colitis showed incontinence in 20.8% of them, and wearing pads during the day was required in 7.6% (15). Long-term outcomes in patients with TCA+ who mostly underwent the Soave or Duhamel procedure showed that 47.6% experienced soiling, while only 52.4% had a normal bowel movement (10). Considering these data, RPC-IPACA is not inferior to the Soave–Duhamel procedure.

Whether proctocolectomy and ileal anal anastomosis should be performed with J-pouch anastomosis is controversial. J-pouch ileoanal canal anastomosis for ulcerative colitis and familial adenomatous polyposis had consistently shown lower stool frequency and better continence rates than straight ileoanal anastomosis; however, these differences were minor, and pouchitis was higher in J-pouch anastomosis (16). These data were collected from patients aged 15 ± 7 years (mean ± standard deviation); therefore, it could not be adopted for patients with HD with a median age of 5 months at radical surgery in this study. Pouchitis occurs in approximately 50% of ulcerative colitis cases, which is characteristic of ulcerative colitis (17); however, it rarely occurs in patients with HD (18). However, in a few neonates and infants, RPC-IPACA for TCA+ showed better functional outcomes (14). We adopted J-pouch ileoanal canal anastomosis rather than straight ileoanal anastomosis because pouchitis is rarely observed in patients with HD, and lower stool frequency prevents buttocks dermatitis, which is advantageous, particularly in neonates and infants who cannot hold back defecation. Another controversial point is the presence or absence of diverting ileostomy. Previous reports show that RPC without diverting ileostomy is effective in approximately 15%–25% of cases (19, 20), and most surgeons opt for protective ileostomy because diverting ileostomy can lower pelvic infectious complications (21). However, in some patients, RPC without diverting ileostomy had similar surgical outcomes in terms of complications as RPC with diverting ileostomy (22). Therefore, we opted for RPC-IPACA without diverting ileostomy because our pediatric patients were in good general condition and could recover well after surgery. Second, we devised a surgical procedure to reduce complications, as described below.

There were three points to prevent peri-and postoperative complications in our RPC-IPACA. First, Lugol's iodine staining was performed. We previously reported that Lugol's iodine staining technique is useful for visualizing the upper margin of the surgical anal canal (Herrmann line) (3). However, misdiagnosing the resected line can cause the aganglionic muscular cuff to remain when resected proximally or injure the anal sensation when resected distally. Therefore, we believe that an appropriate incisional line can reduce the risk of postoperative bowel dysfunction. Second, ICG fluorescence angiography was performed. Poor anastomotic perfusion is a risk factor for anastomotic leakage and poor bowel movement (23). Visual inspection of the resected margins is conventionally used to evaluate anastomotic perfusion; however, it is unreliable in some cases (24). When pulling the J-pouch through the anal canal without tension was impossible, we cut the mesenteric artery to pull it through after examining the J-pouch perfusion using ICG fluorescence angiography. We also used ICG fluorescence angiography to examine the final anastomotic perfusion at the IPACA. Although whether ICG effectively prevents anastomotic leakage remains unclear, ICG can show insufficient perfusion at the resection margin in colorectal surgery (25), thus allowing better anastomosis of the perfusion area. Therefore, we decided that ICG fluorescence angiography within 1 min after ICG injection was appropriate perfusion. This is because the median time to visualize ICG fluorescence in the anastomosis was 29 s (26) and fluorescence intensity showed the highest intensity of approximately 60 s after ICG injection (27). ICG has been used for many decades; however, it has demonstrated its safety with few side effects (28). Because it is easy and safe, we decided that ICG should be used for RPC-IPACA. Third, reduced-port surgery was performed. A single-incision laparoscopic surgery technique has been reported; however, we adopted RPC-IPACA. Therefore, only laparoscopic surgery was not used to complete all manipulations, and laparotomic procedures were required, particularly for creating J-pouches. In our procedure, we resected the bowel from the ileum to the sigmoid colon through the ileostomy incision site or umbilicus Benz incision, followed by creating a J-pouch. We dissected the rectum below the peritoneal reflection *via* laparoscopic surgery, which provided better visualization of pelvic structures, thus making sphincter-and pelvic nerve-preserving surgery feasible. The addition of laparotomy through a small incision contributed to a shorter operative time. Furthermore, there are also cosmetic advantages (Figure 1C), including less pain and invasion and rapid recovery, compared with open or multi-port procedures (29).

Previous studies show that some patients with HD require a redo operation. Indications for re-operation include anastomotic bleeding, bowel obstruction, septum reformation, anorectal stricture, and persistent symptoms such as constipation and recurrent enterocolitis (30, 31). A meta-analysis shows that residual aganglionosis and transition-zone bowel are underlying causes of persistent bowel symptoms in one-third of patients with HD requiring redo operation (30). These persistent bowel symptoms possibly result from partial obstruction due to aganglionic bowel and/or ischemia and internal sphincter spasm due to invasive surgical damage and/or ischemia. Lugol's iodine staining contributes to resecting the adequate line without remaining aganglionic muscle cuff in all cases. This technique helps to prevent residual aganglionosis at the distal intestine/colon and excessive damage to the pelvic plexus; hence, it can decrease persistent bowel symptoms. Another risk of recurrent enterocolitis after HD surgery was significantly increased by anastomotic complications (32). Anastomotic complications, including anastomotic leakage and stricture, are caused by various factors; however, inadequate perfusion is considered one of the main risk factors (33). ICG fluorescence angiography is a possible tool for evaluating anastomotic perfusion, although no consensus has been reached on whether it actually decreases anastomotic complications. One definite disadvantage of ICG is not a quantitative evaluation but a qualitative evaluation. If the quantitative evaluation of anastomotic perfusion using ICG is possible, ICG could more precisely evaluate whether there is adequate perfusion or not, significantly decreasing anastomotic complications. Therefore, we decided ICG fluorescence angiography within 1 min after ICG injection was appropriate perfusion. Moreover, confirming that J-pouch has a certain amount of blood flow contributes to the lack of complications with anastomosis and the J-pouch.

This study had some limitations. First, this study had a small sample size; therefore, some complications may have been missed. Second, a long-term follow-up was not performed. Therefore, evaluation of long-term complications is essential for appropriately assessing our procedure.

In conclusion, our procedure, which is characterized by reduced-port surgery combined with Lugol's iodine staining and ICG fluorescence angiography, was safe, feasible, and cosmetically acceptable for short-term results. Therefore, long-term follow-up is required to evaluate the postoperative bowel function of our procedure.

## Data Availability

The raw data supporting the conclusions of this article will be made available by the authors, without undue reservation.
